# Photosynthetic Plasticity and Stomata Adjustment in Chromosome Segment Substitution Lines of Rice Cultivar KDML105 under Drought Stress

**DOI:** 10.3390/plants12010094

**Published:** 2022-12-24

**Authors:** Narawitch Lertngim, Mathurada Ruangsiri, Suparad Klinsawang, Pimpa Raksatikan, Burin Thunnom, Meechai Siangliw, Theerayut Toojinda, Jonaliza Lanceras Siangliw

**Affiliations:** 1 National Center for Genetic Engineering and Biotechnology (BIOTEC), National Science and Technology Development Agency (NSTDA), Thailand Science Park, Phahonyothin, Khlong Nueng, Khlong Luang, Pathum Thani 12120, Thailand; 2 Department of Agronomy, Faculty of Agriculture at Kamphaeng Saen, Kasetsart University, Kamphaeng Saen, Nakhon Pathom 73140, Thailand

**Keywords:** drought, KDML105, phenomics, photosynthesis, plastic response, stomata density, stomata size, adaxial and abaxial leaf surface

## Abstract

The impact of increasing drought periods on crop yields as a result of global climate change is a major concern in modern agriculture. Thus, a greater understanding of crop physiological responses under drought stress can guide breeders to develop new cultivars with enhanced drought tolerance. In this study, selected chromosome segment substitution lines of KDML105 (KDML105-CSSL) were grown in the Plant Phenomics Center of Kasetsart University in Thailand under well-watered and drought-stressed conditions. Physiological traits were measured by observing gas exchange dynamics and using a high-throughput phenotyping platform. Furthermore, because of its impact on plant internal gas and water regulation, stomatal morphological trait variation was recorded. The results show that KDML105-CSS lines exhibited plasticity responses to enhance water-use efficiency which increased by 3.62%. Moreover, photosynthesis, stomatal conductance and transpiration decreased by approximately 40% and plant height was reduced by 17.69%. Stomatal density tended to decrease and was negatively correlated with stomatal size, and stomata on different sides of the leaves responded differently under drought stress. Under drought stress, top-performing KDML105-CSS lines with high net photosynthesis had shorter plant height and improved IWUE, as influenced by an increase in stomatal density on the upper leaf side and a decrease on the lower leaf side.

## 1. Introduction

Perhaps the most significant issue facing modern agriculture is climate change. Climate change negatively affects crops due to changes brought about by temperature and precipitation [1]. By 2050, the predicted worldwide population will be approximately ten billion people. As a result, doubled global crop production will be required to meet rising food demand. Under predicted climate change scenarios, approximately 50% of people will face water shortages for consumption, with subsequent water deficits in agriculture [2,3,4]. The incidence and magnitude of drought has increased due to climate change which has limited crop production in both quantity and quality [5,6]. Drought is a major issue in Thailand, impeding rice production, which is an important cash crop for the country [7]. Drought stress can affect any stage of rice growth and may thus cause reductions in yield [8]. Although there are irrigation systems for rice cultivation, around 50% of the Thai rice crop is rainfed. Rainfed cultivation is significantly needed as a natural environment and an important ecosystem for rice production [9]. According to research, precipitation in Thailand decreased for six consecutive years, from 2010 to 2016, and rice yield losses were estimated to be 55–68% lower under drought stress [10,11]. This situation necessitates an attempt to develop drought-tolerant cultivars based on knowledge of their physiological mechanisms under drought stress. 

Rice naturally needs to develop strategies to respond effectively to drought. There is a wide range of plant phenotypes that display phenotypic plasticity in response to environmental cues. Understanding phenotypic plasticity may aid in the prediction of plant mechanisms under drought stress and may serve as breeding guidelines [12]. Such mechanistic changes are revealed by physiological and morphological responses under water stress [13]. Since water is an essential component of photosynthesis, any water deficit will directly affect this metabolic process and lead to an abnormal rate of photosynthetic characteristics in plants [14]. As a result, plants need proper gas exchange regulation to function under stressful conditions. This is mediated by microscopic structures called stomata, consisting of a pair of guard cells surrounding a central pore. Stomata regulate the uptake of carbon dioxide into the internal tissues and release water to the environment [15,16]. Moreover, plant temperature regulation and fluid flow are also governed by these structures [17,18]. Stomata are found on both the abaxial and adaxial sides of rice leaves. 

Changes in the surrounding environment cause changes in stomatal density and size [4]. When water becomes scarce, for example, signals such as decreased hydraulic conductivity and increased abscisic acid (ABA) appear, causing guard cell turgor pressure to decrease, resulting in decreased stomatal aperture and stomatal conductance (g_s_) [4]. This morphological change might increase or decrease physiological activity in rice. External signals perceived by mature leaves can also trigger systemic responses that moderate stomatal development on the new leaf epidermis, resulting in stomatal patterning changes [4]. These plastic modulations of stomatal number and size allow plants to adjust their stomatal pore area in response to their surroundings, affecting their maximum and minimum gas exchange [4]. Increased stomatal density, for example, may increase photosynthetic rate and stomatal conductance [19] and the increased rate of photosynthesis caused by gas diffusion changes. A positive correlation between stomatal density, photosynthetic rate, and stomatal conductance is usually found [20]. A further consideration is that adaxial and abaxial stomata might respond differently to each other. These limited information on the impact of stomatal morphological traits on plant physiology and productivity leads to the unrevealed mechanism under drought stress. 

Khao Dawk Mali 105 (KDML105) is an important Thai jasmine rice cultivar planted in rainfed lowland areas of Northeastern Thailand. This area often faces drought stress during the cultivation season leading to a loss in productivity. To understand the mechanism of drought tolerance, chromosome segment substitution lines (CSSL) of KDML105 carrying quantitative loci (QTL) associated with drought stress located on chromosomes 1, 3, 4, 8, and 9 were developed [21,22]. Photosynthesis, gas exchange and stomatal morphology and behavior are responses related to drought tolerance. We used KDML105-CSSLs in this study to investigate their physiological responses and adaptations on stomatal morphology in a greenhouse under well-watered and drought stress conditions, and we identified good-performing KDML105-CSSLs under drought stress based on physiological response and stomatal morphology adjustment. We hypothesized that this cultivar maintains photosynthetic performance by stomatal morphology adjustment. This understanding will help to improve our knowledge of the physiological mechanisms of rice under drought stress and help to design breeding programs and optimal experimental procedures.

## 2. Results

### 2.1. Drought Responses of KDML105-CSSLs Lines

The physiological and stomatal traits of 10 KDML105-CSSLs were investigated under well-watered and drought stress conditions. For drought stress treatments, water was drained starting at 49 DAS until the end of the experiment. Soil moisture was measured (Figure S1), and it dropped by more than 60% in 20 days, indicating severe drought stress [23]. We also calculated the drought stress plasticity of each trait. (Table 1 and Table S1). 

For all physiological traits, we found highly significant differences among treatments. Overall, variation within each condition, including plasticity, was not found. In drought stress conditions, however, there was significant variation among lines in stomatal conductance (g_s_) and transpiration rate (E) (*p* < 0.05). Under irrigated conditions, KDML105-CSS lines (including their donors) had higher rates of net photosynthesis (P_n_), stomatal conductance, and transpiration rate than under drought stress. The three mentioned traits were reduced under drought stress by approximately 38% (Table 1 and Table 2). 

The intrinsic water-use efficiency (IWUE) differed considerably from the previous traits. There was a 3.62% increase in the mean IWUE under drought stress (Table 1). Another trait describing photosynthesis, maximum PSII quantum yield (QYmax), displayed the same pattern as net photosynthesis rate. However, average QYmax decreased by 10% under drought stress, which was less than the 38% decrease in net photosynthesis rate (Table 1 and Table 2). 

Genetic variation was significantly associated with plant height under drought stress. Plant height averaged 695.33 mm in well-watered conditions and 568.34 mm in drought-stressed conditions, respectively. A decrease of 17.7% was observed for plant height under drought conditions (Table 1 and Table 2). 

Stomata morphological traits were collected on both abaxial and adaxial leaf surfaces. Two traits showed variation under different treatments: upper stomatal guard cell length (UP_GCL) and lower stomatal guard cell width (LOW_GCW). However, no differences in stomatal traits were found between lines tested under well-watered, drought-stressed, or plasticity conditions (Table 1 and Table 2; Table S1).

The stomatal density on the upper side was lower than for the lower side in both well-watered and drought-stressed conditions. Moreover, in drought stress, the stomatal density decreased marginally on both leaf sides, accounting for 1.99% and 1.07% for adaxial and abaxial leaf surfaces, respectively. 

There was a slight change in guard cell length and width under drought stress. In normal conditions, guard cell size did not differ, whether on the abaxial or adaxial sides. Guard cells on adaxial leaves increased in length and decreased in width by 7.13 and 2.06%, respectively, while both the length and width of guard cells located on abaxial leaves decreased by 1.67 and 10.17%, respectively (Table 1 and Table 2). The guard cell length was used to calculate the maximum area of the open stomatal pore (UP_ Amax, LOW_ Amax). UP_Amax was slightly higher than LOW_Amax in normal conditions, at 31.09 um^2^ and 29.94 um^2^, respectively. However, responses under drought stress for UP_Amax and LOW_Amax were different. There was a 15.29% increase in UP_Amax, while LOW_Amax slightly decreased by 2.74% (Table 1 and Table 2). 

### 2.2. Relationship among the Evaluated Parameters

**Figure 1:**
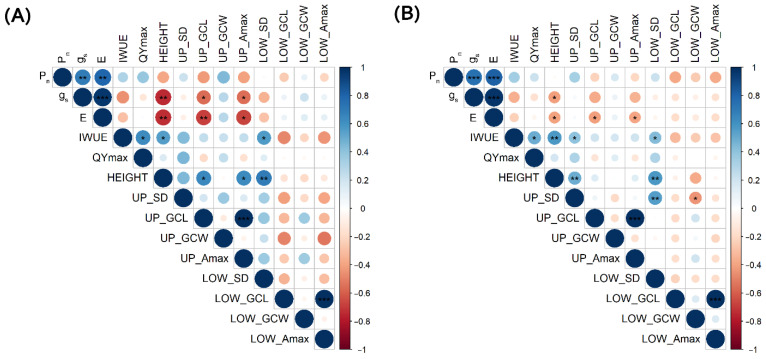
Correlation matrix for among physiological and stomatal morphological traits (**A**) under drought stress and (**B**) plasticity in KDML105-CSSLs. Positive and negative correlations are illustrated in blue and red circles, respectively. Color shade and circle size are proportional to the correlation coefficients, with their values shown in the color intensity bar. Significance level is indicated as * (*p* < 0.05 ‘*’, *p* < 0.01 ‘**’, *p* < 0.001 ‘***’); Net photosynthesis rate (P_n_); Stomatal conductance (g_s_); Transpiration rate (E); Intrinsic water-use efficiency (IWUE); Maximum PSII quantum yield (QYmax); Rice height (HEIGHT); Upper stomatal density (UP_SD); Upper guard cell length (UP_GCL); Upper guard cell width (UP_GCW); Upper maximal pore area (UP_Amax); Lower stomatal density (LOW_SD); Lower guard cell length (LOW_GCL); Lower guard cell width (LOW_GCW); Lower maximal pore area (LOW_Amax).

Strong positive correlations among net photosynthesis, stomatal conductance, and transpiration rate in drought stress conditions and drought stress plasticity were observed (Figure 1). IWUE was correlated with net photosynthesis rate in drought stress, while there is no correlation in plasticity (Figure 1).

In addition, the net photosynthesis rates under drought condition had weak but insignificant correlations (Figure 1) with QYmax, whereas QYmax was positively correlated with IWUE under the plasticity group of traits.

Correlations between plant height and stomatal conductance, transpiration rate, and IWUE were also found. Plant height was negatively correlated with stomatal conductance and transpiration rate but positively correlated with IWUE. The negative correlations under drought stress were stronger than for plasticity (Figure 1).

A moderately positive correlation between the two stomatal densities was shown in drought stress conditions and plasticity (Figure 1). Moreover, there was a positive correlation between stomatal density and IWUE under drought stress plasticity, while the abaxial side showed a higher correlation than the adaxial side. Meanwhile, the densities were also positively correlated to plant height (Figure 1).

There was a negative correlation between upper guard cell length and transpiration rate (plasticity traits) (Figure 1). UP_ Amax was found correlated with the transpiration rate under drought plasticity (Figure 1).

### 2.3. Principal Component Analysis (PCA)

**Figure 2:**
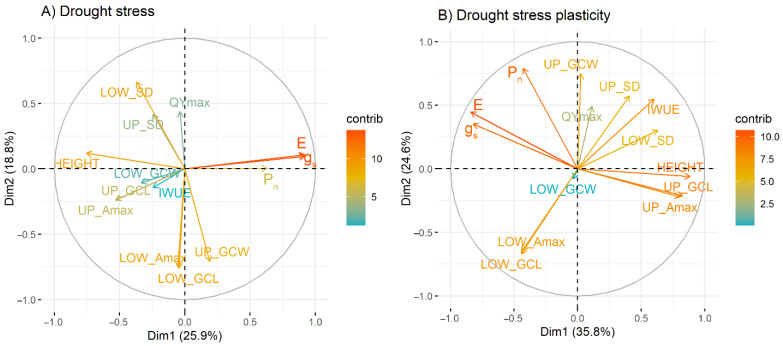
Trait loading scores of the physiological and stomatal traits for each principal component under two conditions; (**A**) drought stress and (**B**) drought stress plasticity.

A principal component analysis (PCA) was performed on all fourteen physiological and stomata morphological traits under drought stress and for drought stress plasticity. Three PCs were considered in both conditions. The three PCs explained almost 60 and 75% of the cumulative variance in all traits under drought stress and for drought stress plasticity, respectively (Figure 2). 

In drought stress, the first component (PC-1) giving 25.87% of the variance which include g_s_, E, HEIGHT and LOW_GCW. Both g_s_ and E were in a similar direction, while other traits in PC-1 are in the opposite direction (Table S2). Meanwhile, UP_SD, UP_GCW, LOW_SD, LOW_GCL and LOW_Amax contributed to the second component (PC-2), accounting for 18.76%. Both stomatal densities followed the same direction, while the others showed the opposite, and contained negative loadings scores. P_n_, IWUE, QYmax, UP_GCL and UP_Amax contributed to the third component (PC-3), with approximately 14.77%. All traits were positively correlated to each other. 

For drought stress plasticity, 35.81% of variance was on PC-1 which include g_s_, E, HEIGHT, UP_GCL, UP_ Amax, and LOW_SD. Both g_s_ and E had a similar direction, while their direction was opposite of the other traits in PC-1. PC-2 consisted of P_n_, IWUE, UP_SD, UP_GCW, LOW_GCL, and LOW_ Amax, giving 24.57% of variance. Almost all traits gave the same direction except for LOW_GCL and LOW_Amax, which had negative loading scores. Only two traits, QY_max and LOW_GCW, constituted PC-3, accounting for 13.90% of the variance. These two traits were negatively correlated to each other (Figure 2 and Table S2).

Overall, the pattern was almost similar in both drought stress and for drought stress plasticity.

### 2.4. Bulk Analysis

**Figure 3:**
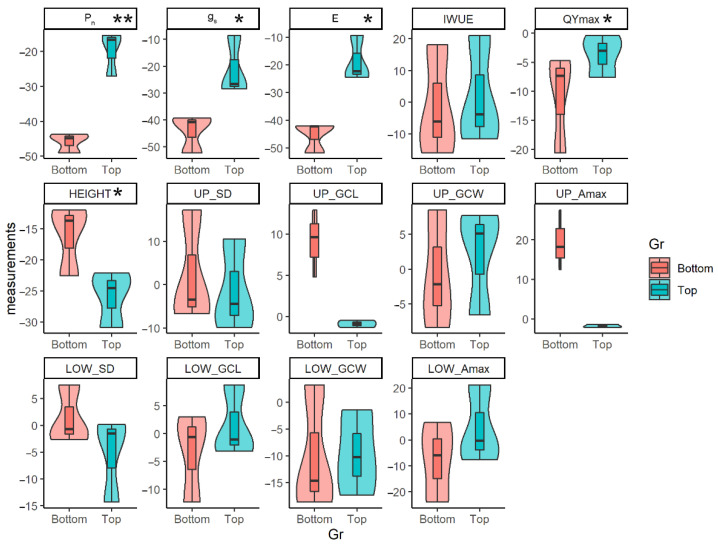
Violin plots of physiological and stomatal traits in drought stress plasticity between two groups of rice; the top (best-performing lines) and bottom (worst-performing lines) groups were represented in pink and blue, respectively (*p* < 0.05 ‘*’, *p* < 0.01 ‘**’).

Bulk segregant analysis was used to select lines that performed good and bad under drought stress. Net photosynthesis rate was chosen as the selection trait since it directly affected biomass and yield production. Six out of ten KDML105-CSS lines were selected. The first three lines performed photosynthesis well in drought stress and have the highest drought stress plasticity. These three lines were CSSL62, CSSL28, and CSSL136. In contrast, the other three lines, namely CSSL119, KDML105, and CSSL29, displayed the lowest photosynthetic plasticity in (Figure 3).

Drought stress plasticity of the selected lines was analyzed using a t-test to investigate dynamics of different photosynthesis mechanisms among the top three best-performing and bottom three worst-performing lines. Violin plots clearly show that net photosynthesis rate was significantly different between these two groups (*p* < 0.01) (Figure 3). Moreover, there was a significant difference in stomal conductance and transpiration rate (*p* < 0.05). The lines which performed well in photosynthesis still showed better plasticity in g_s_ and E than the bottom group (Figure 3). 

The means of maximum PSII quantum yield (QYmax) from the two groups was also significantly different (*p* < 0.05). The top group had higher QYmax drought stress plasticity than the bottom group. In contrast, the bottom group had high plasticity in plant height. Although there was no significant difference in intrinsic water-use efficiency (IWUE) between the top and the bottom group, the top group tended to have a higher IWUE in terms of plasticity (Figure 3).

There were no significant changes in any stomata morphological traits. However, the top group had higher upper stomatal density and upper guard cell width, and the stomatal density in the lower surface tended to be lower than for the upper side. Meanwhile, the upper guard cell length and the upper maximum area of open stomatal pore plasticity were high in the bottom group (Figure 3). 

## 3. Discussion

Rice uses phenotypic plasticity as one of its physiological mechanisms to survive drought stress. Stomata play an important role in gas exchange, which has a direct effect on traits, such as photosynthesis, stomatal conductance, and transpiration [24]. In this experiment, ten selected KDML105-CSS lines showed a highly significant difference in almost all physiological traits (P_n_, g_s_, E, and QYmax) between well-watered and drought stress conditions. However, only g_s_ and E were significantly different among lines in drought stress. Moreover, their physiological performance based on plasticity displayed a similar pattern. Around a 40% decrease was found in net photosynthesis rate, stomatal conductance, and transpiration rate. Furthermore, there were highly significant correlations among these three parameters in both drought stress and drought stress plasticity (*p* > 0.01), indicating relationships in physiological mechanisms that have been previously identified [25]. In contrast, the decrease in the maximum PSII quantum yield (QY_max) only accounted for 9.8%, which was a one-fold decrease compared to the three other physiological traits. Similar to soybean [26], water stress decreased the maximum PSII quantum yield and was found to contribute to P_n_ under stress, just like P_n_ and QY_max sharing variations in PCA in this study. Under drought stress, rice attempted to conserve water by lowering stomatal conductance, as found in the study of Caine et al. [27], with the potential trade-off of reducing carbon assimilation. Moreover, we found that water-use efficiency slightly increased by 3.62% in the experiment, indicating altered drought stress performance.

According to the function of stomata, the microscopic pore plays a vital role in gas exchange, which directly governs physiological mechanisms inside plants [18,24,25]. We expected that the stomatal morphological traits would be modified to maintain internal activity in plants in a stressed environment. However, there was no significant difference in these traits, except for guard cell length at the adaxial side and width at the abaxial side. The guard cell grew 7.13 percent longer on the adaxial side. In contrast, the length was shortened by 1.67% on the abaxial side. It is surprising to observe an increase in guard cell length in the adaxial leaf surface because it is typical for the guard cell to decrease in size upon encountering water stress [28]. Similarly, there was a decrease in guard cell width, but it was noticeable on the abaxial side, which was 10.17%. In contrast, only 2.06% of the width was reduced on the adaxial side. The change in stomatal size led to the change in pore area. There was an increase in upper stomatal maximum pore area (UP_Amax) and a decrease in lower stomatal maximum pore area (LOW_Amax). An increase in UP_Amax contradicts the finding that smaller stomata can efficiently enhance photosynthesis and stomatal conductance, leading to higher yields [29]. However, some *O. sativa* did not show this modification of stomata size. Smaller stomata do not always provide advantages in photosynthesis, according to Zhang et al. [30], because smaller stomata correlate with lower CO_2_ concentrations in leaves [30,31,32,33,34]. In terms of stomatal density, there was a small decrease on both sides. The adaxial and abaxial stomatal densities decreased by 1.99 and 1.07%, respectively. Although there was no significant correlation, the abaxial leaves showed an inverse relationship between stomatal density and stomatal size. This negative relationship between the two traits has been investigated in response to environmental fluctuations, showing the same relationship as found in our experiment [35,36,37]. Franks and Beerling [31] suggested that smaller stomatal sizes and higher stomatal densities are associated with higher maximum stomatal conductance to water. This finding contradicts previous findings that rice reduces stomatal density to maintain water use efficiency [16,28,38]. Hence, KDML105-CSSLs tended to decrease their stomatal density on both sides. They also decreased their guard cell width on both sides and guard cell length on the abaxial side, while guard cell length on the adaxial side significantly increased. The change on the abaxial side was similar to previous findings and likely promotes water conservation [37]. These different plasticity dynamics indicate distinct responses of stomata on different sides of the leaves [39,40].

Water-use efficiency had a strong positive correlation with net photosynthetic rate under both drought and non-drought conditions, but it was not significant for drought stress plasticity. From the plasticity results, although KDML-CSSLs lines maintained their IWUE, their net photosynthetic rate dramatically decreased by almost 40%. This indicates that there are other factors governing IWUE maintenance apart from photosynthesis [41,42]. 

Changes in physiological mechanisms and in stomatal morphology affected rice growth. Height was significantly reduced under drought stress. Moreover, there was variation in height, which is negatively correlated to stomatal conductance and transpiration rate. This relationship could explain the plastic modification of rice to enhance water transport abilities under drought stress since tall plants limit stomatal conductance, which leads to insufficient water for metabolic processes. Thus, rice can enhance their IWUE, which was shown by a significant positive correlation with height in this experiment [42,43,44,45]. The decline in height positively correlated to a decrease in stomatal density on both the adaxial and abaxial sides. This supports the view that decreases in stomatal density help to prevent water loss and maintain stomatal conductance functioning [43,44].

For both drought stress and drought stress plasticity, PCA revealed that stomatal conductance, transpiration rate, and rice height are always together in PC-1. Both stomatal conductance and transpiration rate showed a negative relationship with plant height. This result suggests that stomatal conductance had better performance when rice height was short [45,46], whereas net photosynthesis and water-use efficiency were contained together in PC-3 in both drought stress and drought stress plasticity conditions with the same polarity. This suggests that water use efficiency could be increased by improving carbon assimilation [47,48]. Stomatal traits such as upper stomatal density, upper guard cell width, lower guard cell length, and lower maximal pore area were combined in PC-2 under both drought stress and drought stress plasticity. They were also classified as drought stress plasticity in the same PC as net photosynthesis and water use efficiency. This supports the role of stomata adjustment in water conservation and among physiological mechanisms [49,50].

The physiological mechanisms inducing drought tolerance in KDML105-CSSLs were investigated using bulk analysis. This statistical approach selected and distinguished between the highest and lowest performing lines in net photosynthesis rate plasticity. Each group contained three KDML105-CSSL lines and was defined as the top (best-performing) and bottom (worst-performing) groups. Lines with a high value of plasticity meant a positive change or a slightly negative change in photosynthesis during drought stress. In the top group, there was noticeably high plasticity in photosynthesis. Moreover, other physiological traits, such as stomatal conductance, transpiration rate, and maximum PSII quantum yield, also displayed the same pattern, such that the top group performed well in those traits and was significantly different from the bottom group. Although the water-use efficiency in the top group was higher than the bottom group, it was not statistically different. This is because all lines tried to maintain water usage under stressful conditions, but at different efficiencies. At the same time, rice height in the top group with high photosynthesis was noticeably shorter than in the bottom group, correlating with previous work [45,46]. On the adaxial side, the stomatal density of the top group showed an increasing trend, while the guard cell length and stomatal pore showed the opposite relationship. There was no obvious difference in variation on the abaxial side. This suggests that upper and lower stomata responded differently to drought stress [51]. 

## 4. Materials and Methods

### 4.1. Plant Materails and Growth Conditions

Ten KDML105-CSSLs showing tolerance under drought stress in field conditions were selected for this experiment. The KDML105-CSS lines were developed using the backcross breeding method with marker-assisted selection [22,23]. The seeds were obtained from the Innovative Plant Biotechnology and Precision Agriculture (APBT). KDML105-CSSLs (CSSL26; CSSL28; CSSL29; CSSL37; CSSL54; CSSL62; CSSL119; CSSL123; CSSL128; and CSSL136), KDML105, DH103, and DH212 seeds were incubated at 50 °C for five days. The seeds were cultivated in seed germination cups for a week. Germinated seedlings were transplanted to square plastic pots (16 × 16 cm top, 11 × 11 cm bottom and 20 cm height). The pots were placed using a completely randomized design (CRD) in the open greenhouse at Kasetsart University, Kamphangsaen Campus, Nakhon Pathom, Thailand. The soil type used in this experiment was similar to rice fields within the university, which have a clay form (65.7% clay, 23.3% silt, and 11.0% sand). Each pot contained 6 kg of soil. Water was added, and 50% soil moisture was maintained and measured by a Soil Moisture Meter (Lutron PMS-714 SOIL Moisture Meter IP65, Lutron Electronic Enterprise Co., Ltd., Taipei, Taiwan). The greenhouse had natural ventilation so that the atmosphere, temperature, and humidity represented field conditions. Weeds were controlled manually, and insects were sprayed with insecticides at 55 days after sowing (DAS). Fertilizer formulas 16-8-2 and 46-0-0 were mixed and applied at a rate of 1 g/pot (0.31 g N, 0.04 g P and 0.01 g K) at 18 and 32 DAS. The plants were continuously treated and then moved and placed on the conveyor at the Plant Phenomics Center for plant phenotyping at 46 DAS. The center also featured natural ventilation as described above. The drought stress began at 49 DAS, when plants were at the tillering stage, by withholding watering until the end of the experiment at 64 DAS, while there was always 500 mL of water in the control plots. 

### 4.2. Physiological Traits

Physiological measurements were performed at 50, 53, 57, and 60 DAS. The LI-6400 XT Portable Photosynthesis System (LI-COR Biosciences, Lincoln, NE, USA) was used to collect gas exchange data. In the leaf chambers, the light intensity was set at 1000 µmol^−2^ s^−1^ PAR. The leaf temperature was set at 27 °C, which was equal to the Plant Phenomics Center’s temperature. Atmospheric CO_2_ levels were permitted. Measurements were performed on a second fully expanded leaf. Leaf measurements took approximately 2 min. The width of the leaves was collected to standardize trait values afterward. Three traits were directly obtained using LI-COR, namely net photosynthesis (P_n_), stomatal conductance (g_s_), and transpiration rate (E), while intrinsic water-use efficiency (IWUE) was derived from the ratio of P_n_ to g_s_. 

### 4.3. Plant Phenotyping

Plant phenotype was analyzed at the Plant Phenomics Center, Rice Research Center, Kasetsart University, Kamphangsaen Campus, Nakhon Pathom, Thailand. The PlantScreenTM Phenotyping Systems (Photon Systems Instruments (PSI), Drasov, Czech Republic). The system uses photographs to analyze plant phenotypes. The Chlorophyll Fluorescence Unit and RGB Imaging Unit were further used in the experiment. All plants were photographed five times by each unit throughout the experiment at 49, 54, 56, 61, and 63 DAS. 

The Fv/Fm protocol from the PlantScreenTM Chlorophyll Fluorescence Imaging Unit was used for detecting photosynthetic potential using a FluorCam SN-FC800-200 camera. The distance between the camera and the plant was automatically adjusted. All plants were transferred to dark adaptation for 30 min before fluorescence image capture. Next, 720 × 560-pixel images were acquired, delivering four values of parameters related to photosynthesis. The data included minimum fluorescence in the dark-adapted state (F0), maximum fluorescence in the dark-adapted state (Fm), variable fluorescence in the dark-adapted state (Fv), and maximum PSII quantum yield (Fv/Fm or QY_max). The last parameter, QY_max, was used in this experiment to understand the fundamental mechanisms of photosynthesis under both conditions [52].

The PlantScreenTM RGB Imaging Unit includes a GigE uEye UI-5580SE-C-5 Megapixels QSXGA Camera with a 1/2” CMOS Sensor (IDS, Germany) and 2560 × 1920 resolution pixels. The adjustment of the distance between the camera and plants was automatic and depended on plant height. Images were captured in top and side views. In this experiment, only side-view photographs were used to measure plant height.

### 4.4. Stomatal Traits

Stomata were collected three times, representing three different conditions: before (46 DAS), during (57 DAS), and after (64 DAS) drought stress. Next, nail polish was directly applied on both the abaxial and adaxial sides of the leaves’ central part while avoiding the midrib. The nail polish was then peeled off and pressed on the glass slide to observe stomatal traits.

Upper and lower stomatal densities (UP_SD and LOW_SD) were observed under a microscope at 40x magnification (Olympus BH2-RFCA Fluorescence Microscope). The number of stomata was counted manually in three different locations per sample, and then it was converted to stomatal density, or the number of stomata per mm^2^. After that, the epidermal leaf images were captured by a microscope at 40x magnification (Leica DM500, Leica, Germany) with 1600 × 1200 resolution. On both sides, the guard cell size represented by guard cell length (UP_GCL and LOW_GCL) and width (UP_GCW and LOW_GCW) were measured from the images via the straight line command in the ImageJ program (ImageJ 1.53i). The maximum area of the open stomatal pore (Amax) on two sides (UP_Amax and LOW_Amax) were calculated following Franks and Farquhar’s equation [53,54].

Maximum area of the open stomatal pore:

Amax = π(GCL/4)^2^ (1)

where GCL = guard cell length.

### 4.5. Statistical Analysis

The mean data directly collected from the control and drought stress conditions were used for statistical analysis. Furthermore, drought stress plasticity was also considered. Plasticity was derived from each replication of control and drought stress data according to the formula:

Drought stress plasticity = (DSmean − WWmean)/WWmean(2)

where

DSmean = mean value in drought stress conditions.
WWmean = mean value in well-watered conditions.

A one-way analysis of variance (ANOVA), analyzed by Genstat 21st Edition software, was performed to evaluate the mean, the least significant difference (LSD), and the coefficient of variation (CV) under both conditions. Significant differences in all traits between well-watered and drought stress conditions were analyzed using combined analysis (*p* < 0.05). The correlations between each trait were calculated using Pearson’s correlation coefficient (*p* < 0.05) and analyzed by the ‘corrplot’ R package [55]. The principal component analysis was performed using ‘foctoextra’ R package [56]. While bulk analysis based on plasticity in net photosynthesis rate was analyzed using ‘tidyr’, ‘plyr’, ‘dplyr’ R packages to investigate how plants with a high or low net photosynthesis rate performed in other traits with respect to their physiological mechanisms under drought stress.

## 5. Conclusions

KDML105-CSSL modified their physiological responses to drought stress to maintain photosynthesis. Furthermore, photosynthesis was indirectly influenced by water conservation modifications, which subsequently affected biomass production. In particular, the selected KDML105-CSS lines with high net photosynthesis had their stomatal morphology modified to improve water-use efficiency by increasing density and stomatal pore depth at the adaxial surface while decreasing density and size at the abaxial leaf surface. Aside from stomatal morphology, plant height influences water-use efficiency under stress, and shorter height improves the IWUE as influenced by stomatal morphology changes.

## Figures and Tables

**Table 1:** Statistical summary and the effects of genotype (G effect), treatment (TRT effect) and plasticity on physiological and stomatal traits; well-watered (WW), drought stress (DS). The significance level is indicated as ‘*’ depending on *p*-values (*p* < 0.05 ‘*’, *p* < 0.01 ‘**’).

Traits	TRT	Mean	LSD	Min	Max	CV	G Effect	TRT Effect
P_n_	WW	12.33	3.48	10.33	15.03	13.08	ns	**
	DS	7.49	3.32	6.04	9.4	20.54	ns	
	Plasticity	−37.69	38.82	−58.07	−15.42	−47.69	ns	
g_s_	WW	0.14	0.051	0.112	0.17	16.85	ns	**
	DS	0.083	0.024	0.068	0.106	13.07	*	
	Plasticity	−38.63	32.1	−55.038	−8.497	−38.47	ns	
E	WW	0.0035	0.0012	0.0028	0.0042	15.85	ns	**
	DS	0.0021	0.0006	0.0017	0.0027	13.23	*	
	Plasticity	−38.41	33.01	−51.76	−9.3248	−39.77	ns	
IWUE	WW	89.06	19.05	75.96	106.69	9.9	ns	ns
	DS	90.89	29.4	70.24	121.33	14.97	ns	
	Plasticity	3.62	37.51	−15.97	45.36	479.73	ns	
QYmax	WW	0.7565	0.0711	0.7195	0.7846	4.3517	ns	**
	DS	0.681	0.1257	0.5717	0.7596	8.5444	ns	
	Plasticity	−9.84	17.81	−25.06	−0.2037	−83.81	ns	
HEIGHT	WW	695.33	162.98	632.3	788.7	10.85	ns	**
	DS	568.34	71.81	442.4	675.5	5.85	**	
	Plasticity	−17.69	21.19	−31.77	−0.59	−55.45	ns	
UP_SD	WW	512.16	99.19	455.56	587.65	8.96	ns	ns
	DS	497.1	130.34	453.08	562.96	12.14	ns	
	Plasticity	−1.99	38.04	−14.19	17.2	−885.82	ns	
UP_GCL	WW	12.44	1.8	11.3	13.37	6.69	ns	**
	DS	13.26	1.7	12.55	14.07	5.93	ns	
	Plasticity	7.13	21.17	−1.35	18.22	137.42	ns	
UP_GCW	WW	3.88	0.54	3.55	4.37	6.46	ns	ns
	DS	3.78	0.56	3.55	3.97	6.88	ns	
	Plasticity	−2.06	48.52	−13.7	9.27	−1,087.87	ns	
UP_Amax	WW	31.09	9.43	25.71	35.76	14.05	ns	ns
	DS	35.15	9.8	31.56	39.48	12.9	ns	
	Plasticity	15.29	48.52	−2.19	42.43	146.87	ns	
LOW_SD	WW	654.51	143.81	560.49	706.17	10.17	ns	ns
	DS	642.17	109.9	603.7	676.54	7.92	ns	
	Plasticity	−1.07	24.97	−14.32	18.25	−1,083.73	ns	
LOW_GCL	WW	12.2	1.79	11.33	13.52	6.81	ns	ns
	DS	11.95	2.47	10.06	14.13	9.56	ns	
	Plasticity	−1.67	21.7	−13.71	11.06	−601.25	ns	
LOW_GCW	WW	3.89	0.7	3.59	4.16	8.33	ns	***
	DS	3.47	0.58	3.2	3.95	7.79	ns	
	Plasticity	−10.17	25.11	−18.66	3.25	−114.27	ns	
LOW_Amax	WW	29.94	8.61	25.74	26.26	13.32	ns	ns
	DS	28.73	13.18	19.99	40.6	21.24	ns	
	Plasticity	−2.74	46.64	−25.43	24.53	−788	ns	

G effect, genotype effect; TRT effect, treatment effect; ns, non-significant.

**Table 2:** Mean value of KDML105-CSSLs and parents for the physiological and stomata morphology traits under well-watered (WW) and drought stress (DS) conditions.

LINES	TRT	P_n_	g_s_	E	IWUE	QYmax	HEIGHTT	UP_SD	UP_GCL	UP_GCW	UP_Amax	LOW_SD	LOW_GCL	LOW_GCW	LOW_Amax
CSSL 26	WW	12.40085	0.149529	0.003687	83.2152	0.743846	701	470.3704	12.35515	4.033852	31.19718	606.1728	12.57833	4.018169	31.63711
CSSL 28	WW	13.27751	0.146094	0.003673	90.78073	0.761377	689.5	506.1728	12.85801	3.770606	33.72576	706.1728	12.19166	3.593803	29.94588
CSSL 29	WW	10.82491	0.142655	0.003648	75.96116	0.758405	674.7	455.5556	11.83199	3.799463	28.17341	696.2963	11.49074	4.028734	26.40059
CSSL 37	WW	10.48667	0.13565	0.003459	76.79568	0.746339	652.9	508.642	11.35872	3.947241	25.87774	670.3704	11.78924	3.609668	27.64564
CSSL 54	WW	12.74923	0.154158	0.003859	83.00898	0.768806	679.2	480.2469	12.53246	3.54823	31.14717	560.4938	13.52053	4.164308	36.36476
CSSL 62	WW	10.33348	0.122282	0.00291	84.72687	0.762349	742.1	500	13.37501	3.918357	35.64489	671.6049	11.32786	3.904699	25.73798
CSSL 119	WW	12.04496	0.11365	0.002917	106.6908	0.783779	700.3	517.284	12.46539	3.896942	31.05866	629.6296	11.44258	3.827262	26.01624
CSSL 123	WW	12.43124	0.13139	0.003306	94.62477	0.784559	695.5	579.0123	11.3036	3.660179	25.70711	675.3086	12.11961	3.882777	29.42015
CSSL 128	WW	12.28607	0.136865	0.003385	90.97505	0.759657	653.7	527.1605	11.7867	3.989174	27.62395	698.7654	13.09047	3.80141	34.12915
CSSL 136	WW	10.99383	0.111877	0.002818	97.83279	0.764345	730.6	475.3086	13.34637	3.667335	35.75589	632.0988	12.3559	3.738654	30.72173
DH 103	WW	14.3001	0.17023	0.004185	86.35481	0.724581	632.3	587.6543	13.37404	4.284816	35.69122	655.5556	11.89782	3.913337	28.39954
DH 212	WW	15.03306	0.159582	0.00385	94.90108	0.757108	698.8	529.6296	13.07897	4.372234	33.9856	638.2716	12.895	4.152852	33.75207
KDML 105	WW	13.08381	0.142357	0.003616	91.91278	0.719508	788.7	520.9877	11.99898	3.564857	28.5624	667.9012	11.85631	3.888481	29.01944
CSSL 26	DS	8.535008	0.082756	0.002137	102.8125	0.711213	592.3	464.1975	13.50081	3.725274	36.48579	637.037	11.20324	3.82478	25.07327
CSSL 28 *	DS	9.39688	0.105755	0.002732	86.99507	0.738096	473.5	454.321	12.65097	3.966569	31.79215	603.7037	11.80988	3.54298	27.66314
CSSL 29	DS	6.110094	0.068145	0.001761	89.40127	0.721516	580	530.8642	12.97404	3.711418	33.30658	675.3086	10.06432	3.253083	19.98689
CSSL 37	DS	6.035887	0.070819	0.001739	85.2332	0.739112	568.7	516.0494	13.38775	3.687008	36.50275	634.5679	12.11951	3.726083	29.14734
CSSL 54	DS	8.278924	0.06961	0.001904	121.33	0.747777	675.5	495.0617	14.01411	3.875013	39.29486	655.5556	11.66471	3.446318	27.09667
CSSL 62 *	DS	8.331205	0.081988	0.002115	102.4924	0.759609	576.65	479.0123	13.17886	3.661818	35.03889	661.7284	12.1971	3.199996	30.04031
CSSL 119	DS	6.116743	0.068462	0.001673	89.65746	0.624153	598.5	480.2469	14.06655	3.551428	39.47764	676.5432	11.76777	3.951293	27.56656
CSSL 123	DS	7.60206	0.083981	0.001941	90.58027	0.588482	604	562.963	12.66098	3.750572	31.78102	645.679	11.53657	3.387278	26.64317
CSSL 128	DS	8.062722	0.100935	0.002419	79.96742	0.571675	442.4	453.0864	12.63332	3.72549	31.56042	648.1481	11.833	3.492691	28.0137
CSSL 136 *	DS	8.752824	0.100977	0.002521	85.7431	0.706101	549.2	508.642	13.25533	3.932211	34.7958	622.2222	12.13645	3.292633	29.69243
DH 103	DS	6.061651	0.08681	0.002091	70.24229	0.631991	546.3	551.2346	13.90681	3.696656	38.3	617.284	13.12586	3.230416	34.62566
DH 212	DS	6.858925	0.075496	0.001958	91.05952	0.646964	570.3	466.6667	13.61416	3.928358	36.86224	606.1728	14.13031	3.411843	40.59869
KDML 105	DS	7.218214	0.084343	0.002093	86.09545	0.666582	611.1	500	12.54595	3.868894	31.80701	664.1975	11.78291	3.309272	27.31333

* Top performing lines based on bulk analysis method. Note: TRT—treatment; P_n_—Net photosynthesis rate; g_s_—stomatal conductance; E—transpiration rate; IWUE—intrinsic water use efficiency; QYmax—maximum PSII quantum yield; Height—plant height; UP_SD—upper surface stomatal density; UP_GCL—upper stomatal guard cell length; UP_GCW—upper stomatal guard cell width; UP_amax—maximum area of the open upper stomatal pore; LOW_SD—lower surface stomatal density; LOW_GCL—lower stomatal guard cell length; LOW-GCW—lower stomatal guard cell width; LOW-amax—maximum area of the open lower stomatal pore.

## Data Availability

Data is contained within the article or Supplementary Material.

## Supplementary Materials

The following are available online at https://www.mdpi.com/article/10.3390/plants12010094/s1, Figure S1: The average soil moisture measured during the experiment, starting from 49 days after sowing (DAS) to 63 DAS; The blue and orange lines indicated soil moisture in percent of rice under well-watered (WW) and drought-stressed (DS) conditions, respectively. Table S1 Mean value of KDML105-CSSLs for traits plasticity. Table S2: The physiological and stomatal trait loading scores for each principal component under drought stress plasticity; the bold number indicated the maximum magnitude of loading score among the three PCs.
