# Supplementary material for: Photosynthetic Plasticity and Stomata Adjustment in Chromosome Segment Substitution Lines of Rice Cultivar KDML105 under Drought Stress
Source: Plants (Basel). 2022 Dec 24;12(1):94. doi: 10.3390/plants12010094 (PMC9823560; doi:10.3390/plants12010094)
Supplement: Supplementary file 1 [file plants-12-00094-s001.zip › plants-2040230-supplementary.pdf]

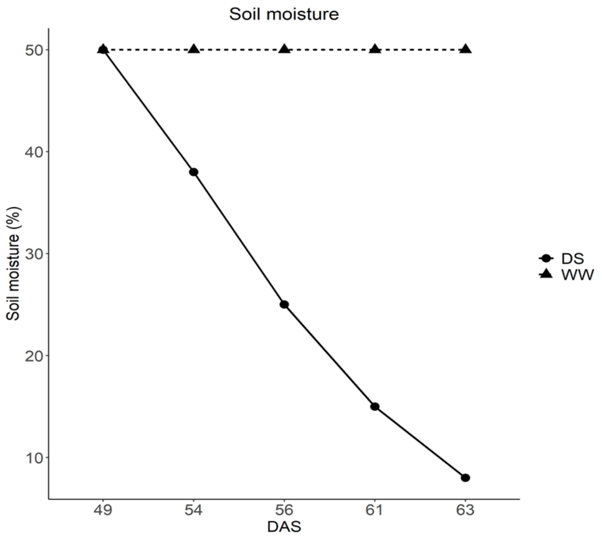

Figure S1. The average soil moisture measured in WW (well-watered) and DS (drought stress) during the experiment. DAS – days after sowing

Table S1 Mean value of KDML105-CSSLs for traits plasticity.

LINES	PHOTO	COND	TRANS	IWUE	QYmax	HEIGHT	UP_SD	UP_GCL	UP_GCW	UP_Amax	LOW_SD	LOW_GCL	LOW_GCW	LOW_Amax
CSSL 26	-31.288	-44.308 4	-41.4583	23.7545	-4.38292	-15.0936	0.399188	9.133961	-6.99145	16.19972	5.127439	-10.8998	-3.21554	-20.6902
CSSL 28	-27.1056	-26.6549	-24.475	-3.88697	-3.04645	-30.9024	-9.90431	-0.90654	5.136294	-2.19272	-14.3238	-3.16861	-1.4493	-7.71278
CSSL 29	-43.6594	-52.2024	-51.7557	18.11423	-4.70759	-11.9842	17.1983	9.637791	-2.17919	18.26353	-2.64199	-12.3451	-18.6608	-24.0242
CSSL 37	-41.0485	-47.1754	-49.3645	11.20877	-0.20365	-12.5829	1.432895	18.21845	-5.6476	42.43372	-4.38014	2.787204	3.180778	5.365834
CSSL 54	-35.0673	-55.0381	-50.8223	45.35693	-2.74111	-0.59235	3.728035	11.97335	9.268469	26.83079	18.24969	-13.7111	-17.2533	-25.4302
CSSL 62	-15.422	-28.4639	-22.256	21.08699	-0.35887	-22.1211	-4.4645	-1.34714	-6.58801	-1.42523	-1.52593	8.739458	-17.3434	21.20408
CSSL 119	-48.9966	-39.3074	-42.0515	-15.9707	-20.5813	-13.6946	-6.74265	12.92369	-8.38483	27.53154	7.600486	2.975328	3.248819	6.716273
CSSL 123	-38.8501	-36.0884	-41.2925	-4.30086	-25.059	-12.8588	-2.36976	13.29023	2.475452	29.45792	-4.23832	-4.79722	-12.627	-9.14668
CSSL 128	-34.339	-25.7086	-28.081	-11.0236	-24.76	-31.7659	-14.1937	7.186382	-6.37431	14.31439	-6.76903	-9.59489	-7.94299	-17.9147
CSSL 136	-16.6967	-8.49677	-9.32485	-11.522	-7.56462	-24.5662	10.52668	-0.47296	7.710485	-1.84076	0.190476	-1.08106	-10.2462	-0.24877
DH 103	-58.0674	-45.4521	-47.1865	-15.4497	-12.6591	-13.4194	-6.15385	3.983787	-13.7045	7.302218	-5.57698	11.06131	-17.5061	24.52755
DH 212	-54.5148	-52.5447	-49.1335	-4.22635	-14.4863	-17.8446	-11.8266	4.297487	-10.0602	9.420382	-4.93616	8.939068	-17.7556	17.64002
KDML 105	-44.8539	-40.7426	-42.158	-6.09543	-7.3474	-22.5361	-3.47323	4.793032	8.501202	12.4977	-0.64092	-0.62337	-14.665	-5.8993

Note: P_n – Net-photosynthesis rate; g_s – stomatal conductance; E – transpiration rate; IWUE – intrinsic water use efficiency; QYmax - maximum PSII quantum yield; Height – plant height; UP_SD – upper surface stomatal density; UP_GCL - upper stomatal guard cell length; UP_GCW - upper stomatal guard cell width; UP_amax - maximum area of the open upper stomatal pore; LOW_SD – lower surface stomatal density; LOW_GCL – lower stomatal guard cell length; LOW-GCW – lower stomatal guard cell width; LOW-amax – maximum area of the open lower stomatal pore

Table S2. Trait loading scores of physiological and stomatal traits for each principal component under drought stress plasticity; Bold number indicated the maximum magnitude of loading score among three PCs.

Trait	Factor loadings					
	Drought stress			Drought stress plasticity		
	**PC-1**	**PC-2**	**PC-3**	**PC-1**	**PC-2**	**PC-3**
P_n	0.33	0.00	**0.46**	-0.19	**0.43**	0.02
g_s	**0.48**	0.06	0.07	**-0.37**	0.19	0.22
E	**0.48**	0.07	0.16	**-0.38**	0.24	0.10
IWUE	-0.13	-0.09	**0.52**	0.27	**0.30**	-0.26
QYmax	-0.02	0.27	**0.34**	0.05	0.27	**-0.41**
HEIGHT	**-0.40**	0.07	0.20	**0.40**	-0.03	-0.20
UP_SD	-0.13	**0.26**	-0.11	0.18	**0.31**	-0.15
UP_GCL	-0.28	-0.15	**0.32**	**0.37**	-0.11	0.32
UP_GCW	0.10	**-0.44**	0.10	0.01	**0.40**	0.09
UP_a_{max}	-0.28	-0.15	**0.34**	**0.36**	-0.12	0.30
LOW_SD	-0.19	**0.41**	-0.16	**0.28**	0.16	-0.09
LOW_GCL	-0.02	**-0.47**	-0.17	-0.20	**-0.36**	-0.31
LOW_GCW	**-0.18**	-0.07	-0.08	-0.02	-0.04	**0.49**
LOW_a_{max}	-0.03	**-0.46**	-0.16	-0.20	**-0.35**	-0.33
Eigenvalues	3.62	2.63	2.07	5.01	3.44	1.95
Variance percent (%)	25.87	18.76	14.77	35.81	24.57	13.90
Cumulative variance percent (%)	25.87	44.63	59.40	35.81	60.39	74.29

Note: P_n – Net-photosynthesis rate; g_s – stomatal conductance; E – transpiration rate; IWUE – intrinsic water use efficiency; QYmax - maximum PSII quantum yield; Height – plant height; UP_SD – upper surface stomatal density; UP_GCL - upper stomatal guard cell length; UP_GCW - upper stomatal guard cell width; UP_amax - maximum area of the open upper stomatal pore; LOW_SD – lower surface stomatal density; LOW_GCL – lower stomatal guard cell length; LOW-GCW – lower stomatal guard cell width; LOW-amax – maximum area of the open lower stomatal pore
